# A Platform Integrating Biophysical and Biochemical Stimuli to Enhance Differentiation and Maturation of Cardiomyocyte Subtypes Derived from Human Induced Pluripotent Stem Cells

**DOI:** 10.3390/jcdd12020056

**Published:** 2025-02-04

**Authors:** Zhonggang Feng, Kota Sawada, Iori Ando, Riku Yoshinari, Daisuke Sato, Tadashi Kosawada

**Affiliations:** Graduate School of Science and Engineering, Yamagata University, Yonezawa 992-8510, Japan; t233012m@st.yamagata-u.ac.jp (K.S.); t243676m@st.yamagata-u.ac.jp (I.A.); t243209m@st.yamagata-u.ac.jp (R.Y.); d_sato@yz.yamagata-u.ac.jp (D.S.); kosawada@yz.yamagata-u.ac.jp (T.K.)

**Keywords:** bioreactor, mechano-electrical stimulation, cardiac differentiation, cardiomyocyte subtypes, three-dimensional culture, ventricular extracellular matrix, subtype aggregation

## Abstract

To enhance the differentiation and maturation of cardiomyocytes derived from human induced pluripotent stem cells, we developed a bioreactor system that simultaneously imposes biophysical and biochemical stimuli on these committed cardiomyocytes. The cells were cultured within biohydrogels composed of the extracellular matrix extracted from goat ventricles and purchased rat-origin collagen, which were housed in the elastic PDMS culture chambers of the bioreactor. Elastic and flexible electrodes composed of PEDOT/PSS, latex, and graphene flakes were embedded in the hydrogels and chamber walls, allowing cyclic stretch and electrical pulses to be simultaneously and coordinately applied to the cultured cells. Furthermore, a dynamic analysis method employing the transverse forced oscillation theory of a cantilever was used to analyze and discriminate the subtype-specific beating behavior of the cardiomyocytes. It was found that myosin light chain 2v (*MLC2v*), a ventricular cell marker, was primarily upregulated in cells aggregated on the (+) electrode side, while cardiomyocytes with faint *MLC2v* but strong cardiac troponin T (*cTNT*) expression aggregated at the ground electrode (GND) side. mRNA analysis using rtPCR and the gel beating dynamics further suggested a subtype deviation on the different electrode sides. This study demonstrated the potential of our bioreactor system in enhancing cardiac differentiation and maturation, and it showed an intriguing phenomenon of cardiomyocyte subtype aggregation on different electrodes, which may be developed into a new method to enhance the maturation and separation of cardiomyocyte subtypes.

## 1. Introduction

The application of cardiomyocytes differentiated from human induced pluripotent stem cells (hiPS-CMs) has shown great promise for both clinical use [1,2] and drug screening [3,4]. However, the challenge of obtaining mature hiPS-CMs limits their clinical application to harnessing only cytokine secretion and nurturing effects, rather than directly reinforcing heart pump function. Additionally, the lack of effective and low-cost methodologies to differentiate and purify cardiomyocyte subtypes compromises the relevance and efficacy of drug screening [4,5].

To enhance hiPS-CM maturation, subtype differentiation, and separation, researchers have explored various techniques to introduce biochemical and biophysical factors into their culture systems. Since mechanical and electrical activities are the most prominent features of heart muscle in vivo, it is straightforward and natural to apply these stimuli to cardiomyocytes or their three-dimensional (3D) constructs cultured in vitro. Several comprehensive reviews and reports discuss the use of electromechanical stimuli in this context [6,7]. Notably, our research group was among the first to investigate the simultaneous application of electrical and mechanical stimuli to 3D cultures of cardiomyocytes [8]. Recently, using dynamic, free-floating culture conditions, 3D cylindrical tissues were created from neonatal rat cardiomyocytes or hiPS-CMs by embedding the cells into a fibrin-based hydrogel, which resulted in significant contractile enhancement [9]. Another study utilized a coculture of hiPS-CMs and hiPS-derived cardiac fibroblasts in a 3D fibrin matrix to form engineered cardiac tissue constructs, subjected to continuous stress by circumferential mounting on nylon tabs. The hiPS-CMs developed a t-tubular system and exhibited calcium handling and contractile kinetics comparable to those of the human ventricular myocardium [10].

The appropriate stem cell response to electrical stimulation, particularly in differentiating into cardiomyocytes, has been demonstrated [11]. Given the role of pulsatile signals in the development of the cardiac syncytium [12], it has been shown that applying pulsatile electrical signals in cardiac tissue engineering can enhance the functional coupling of cells and promote the formation of synchronously contractile tissue constructs, as reported by different research groups [12,13,14].

The materials used for electrodes in bioelectrical stimulation applications must meet biocompatibility requirements. In this regard, stretchable and conductive organic substances have garnered increasing attention [15,16,17]. Poly(3,4-ethylenedioxy-thiophene):poly(styrene sulfonate) (PEDOT/PSS) has been proven to be a flexible and conductive material with good biocompatibility. Furthermore, the addition of graphene flakes into those organic electrodes has been shown to improve electrical conductivity, and graphene may also promote cellular migration [18].

In a 3D culture system, the scaffold plays a crucial role in maintaining the integrity of the 3D construct and facilitating chemical and mechanical interactions with embedded cells. Recently, the use of natural substances derived from the extracellular matrix has shown significant benefits in cardiac tissue engineering, particularly in terms of biochemical stability, enhanced cardiomyocyte maturation, and feasibility for clinical transplantation [19,20,21,22].

The success of the above-mentioned efforts may be attributed to the fact that cardiomyocyte differentiation and maturation do not rely on a single “key node” gene, as in skeletal muscle. Instead, they follow an intertwined network, in which each factor plays its corresponding role in an orchestrated process [23,24,25]. Moreover, each factor appears to exert its enhancing effects relatively independently [21].

Inspired by these achievements, we developed an integrated system that simultaneously applies biophysical and biochemical stimuli to hiPS-CMs. The cells were cultured within biohydrogels made from the extracellular matrix extracted from goat ventricles, housed in elastic culture chambers made from polydimethylsiloxane (PDMS) within the bioreactor. Elastic, flexible electrodes composed of PEDOT/PSS, latex, and graphene flakes were embedded in the hydrogels and chamber’s walls, allowing for the simultaneous and coordinated application of cyclic stretch and electrical pulses to the cultured cells. Additionally, a dynamic analysis method based on the transverse forced oscillation theory of a cantilever was employed to distinguish the subtype-featured beating behavior of cardiomyocytes within the hydrogels. In this paper, we describe the system in detail and report a preliminary experiment to demonstrate the potential of this integrated bioreactor system.

## 2. Materials and Methods

### 2.1. hiPS Cell Culture and Cardiomyocyte Differentiation

The hiPS cell line (RBRC-HPS0002) was purchased from the RIKEN Cell Bank. The hiPS cell culture and differentiation at our lab [26] followed protocols detailed in other prior studies [27,28]. Briefly, hiPS cells were maintained and expanded using feeder-independent culture with mTeSR1 medium (Stemcell Technologies, Vancouver, BC, Canada) in six-well plates (AS ONE, Osaka, Japan) coated with Matrigel (Corning, NY, USA). After expansion, the cells were transferred to tissue culture flasks (Iwaki, Shizuoka, Japan) coated with Matrigel and cultured until 95–98% confluency was achieved.

Cardiomyocyte differentiation was induced by CHIR-IWP2 treatment to modulate the Wnt signaling pathway, marking differentiation initiation as day 0. The resultant hiPS-derived cardiomyocytes (hiPS-CMs) were cultured in serum-free Roswell Park Memorial Institute (RPMI)/B-27 medium (Thermo Fisher Scientific, Waltham, MA, USA) supplemented with 1% 4-(2-hydroxyethyl)-1-piperazineethanesulfonic acid (HEPES). The medium (RPMI plus) was replaced every other day. By day 15, spontaneous beating of the hiPS-CMs was evident under an inverted microscope (Olympus, Tokyo, Japan). Subsequently, hiPS-CMs were harvested for 3D culture in our integrated bioreactor system.

### 2.2. Ventricular Extracellular Matrix (vECM) Extraction and Gel Casting

The protocol for extracting ventricular extracellular matrix (vECM) was adapted from our previously reported method [29]. Briefly, heart ventricles from adult goats were sliced (~2 mm thick), minced into small pieces, soaked in 1 N saline solution (Sigma-Aldrich, St. Louis, MO, USA) for 2 h, and rinsed twice with deionized water (DW) for 15 min each. Tissue pieces were decellularized by stirring in a 1% (*w*/*v*) sodium dodecyl sulfate (SDS) solution (Sigma-Aldrich) in phosphate-buffered saline (PBS) for 4–5 days, followed by a 1% (*w*/*v*) Triton X-100 solution (Wako, Tokyo, Japan) in PBS for 24 h. If necessary, this process was repeated to ensure thorough decellularization.

After decellularization, vECM pieces were rinsed with DW for 3 days, homogenized, and digested with pepsin (≥2500 units/mg, Sigma-Aldrich) in 10 mM HCl (Wako) for 48 h to produce vECM solutions (15–22 mg/mL). The pepsin-to-tissue mass ratio was 1:250. Finally, vECM solutions were adjusted to pH 7.4 and physiological salt concentration (1× PBS) using 1.0 M NaOH and 10× PBS, respectively.

For 3D culture, hiPS-CMs were embedded in hydrogels composed of vECM and collagen (rat origin, BD Biosciences, Tokyo, Japan). Hydrogel mixtures were prepared to final concentrations of 1.5 mg/mL collagen and 0.6 mg/mL vECM. After adjusting pH and salt concentration, a cell suspension containing 2 × 10^6^ hiPS-CMs was added, and the mixture was cast into culture chambers of the bioreactor. Gel formation occurred by incubating in a 5% CO_2_ incubator for 1 h. Each gel had a volume of 0.5 mL and a thickness of 3.0 mm. The 3D culture was maintained in RPMI plus medium with daily medium changes for two weeks [30,31]. Therefore, the differentiation of hiPS cells proceeded in two stages: the first stage, a monolayer differentiation process described in the previous subsection, lasted 15 days; the second stage, the 3D culture process described here, also lasted 15 days. Physical stimuli, as detailed below, were applied throughout the 15 days of the second stage.

### 2.3. Bioreactor System

**Figure 1:**
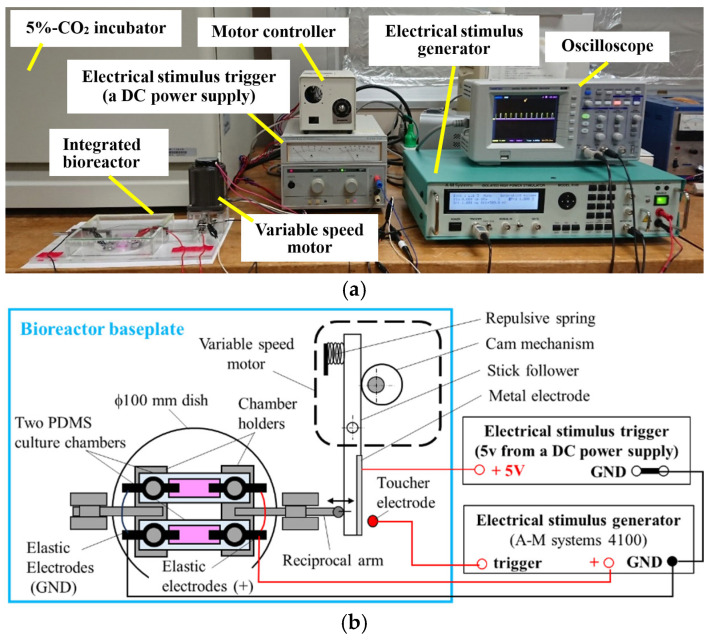
Integrated bioreactor system for 3D culture of hiPS-CMs. (**a**) Photograph of the complete system, designed for 3D culture of hiPS-CMs embedded in vECM-collagen hydrogel within the integrated bioreactor. The bioreactor applies mechanical and electrical stimulation to the hydrogels inside its culture chambers, which are housed in a commercially available 5% CO_2_ incubator. (**b**) Schematic diagram of the system illustrating its operational mechanism, connections between components, and the coordination of mechanical and electrical stimulation via trigger signaling.

**Figure 2:**
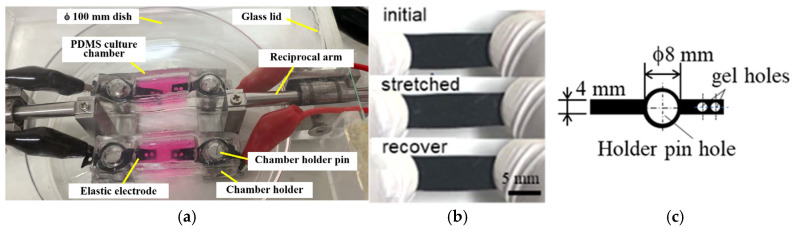
Details of the culture chambers and the electrodes within the bioreactor. (**a**) Photograph showing two culture chambers installed into the bioreactor system. (**b**) Demonstration of the elastic stretchability of the conductive electrode material (PEDOT/PSS-latex-rGO), developed in-house. (**c**) Configuration of the electrodes embedded within the PDMS culture chambers, highlighting their integration for effective electrical stimulation.

Figure 1a shows the integrated bioreactor system, which consisted of two culture chambers, a reciprocal stretch activator (driven by a variable-speed motor), an electrical stimulus generator, a monitoring oscilloscope, and a CO_2_ incubator housing the culture chamber device. As shown in Figure 1b and Figure 2a, culture chambers were fabricated from polydimethylsiloxane (PDMS) and mounted on stainless-steel holders. Each chamber measured 20.0 mm × 8.0 mm × 7.5 mm and featured two end holes for attachment to cylinder pins of the holders. One holder was stationary, while the other was actuated by a reciprocal arm imposing cyclic strain on the PDMS chambers.

Figure 1b illustrates the operational scheme and connections between components of the bioreactor system. The reciprocal arm movement, driven by an eccentric cam mechanism powered by the variable-speed motor, allowed for adjustable stroke distances based on the cam’s eccentric ratio. A metal electrode mounted on the cam’s stick follower contacted a toucher electrode during each stretch cycle, triggering a 5 V trigger signal from a DC power supply to the electrical stimulus generator. This trigger mechanism ensured synchronized mechanical and electrical stimulation of the culture chambers. The timing of the electrical pulse relative to the mechanical stretch could be adjusted by programming a delay for the electrical stimulus generator.

Elastic organic electrodes developed in-house (Figure 2b,c) delivered electrical stimulus to the gels in the culture chambers. These electrodes, based on PEDOT/PSS (1.1% in H_2_O, Sigma: 739324-100G) [32], were rendered stretchable by adding natural latex rubber solution (Kenis: 126-0252) in a 5:1 ratio. To enhance conductivity, 0.05 g of lithium bis(trifluoromethanesulfonyl)imide (Sigma: 449504) and 10.0 mg of reduced graphene oxide (rGO) were incorporated. After drying at room temperature and heating to 130 °C for 10 min, the elastic film was shaped, as shown in Figure 2c. The electrodes exhibited conductivity of 487.4 ± 49.6 S/m (n = 5) and were integrated into the end walls of the culture chambers, with gel–hole sides embedded into the hydrogel. During operation, cyclic stretching of the elastic PDMS chambers and electrodes ensured synchronized application of mechanical and electrical stimuli to the hydrogel-embedded cells.

### 2.4. Measurement of Contractile Force Profile

**Figure 3:**
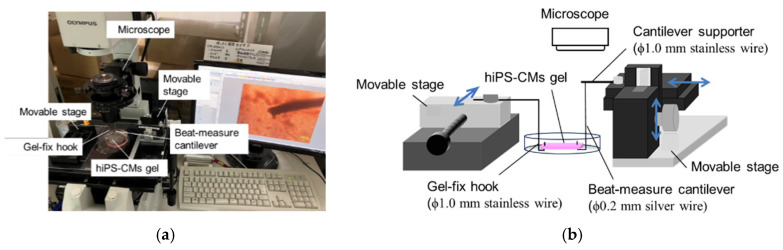
Photograph of device for the beating force measurement (**a**) and its schematic drawing (**b**). Arrows in (**b**) indicate stage-moving directions.

The beating of hiPS-CM hydrogels was analyzed using a vision-based micro-displacement measurement setup developed in our lab (Figure 3a). The hydrogel was detached from the electrodes and removed from the culture chamber into a dish filled with culture medium. The gel’s two ends were hooked onto separate hooks: one, made of a ϕ1.0 mm stainless-steel wire, served as a fixed anchor, while the other, made of a ϕ0.2 mm silver wire, was attached to a ϕ1.0 mm stainless-steel support forming a cantilever probe (Figure 3b). The beating motion at the cantilever’s free end, where it was hooked to the gel, was recorded using an inverted microscope (Olympus IX71) at 60 fps.

To measure the contractile force, the stage supporting the silver wire was adjusted to set different stretch ratios (0 to approximately 20%) relative to the gel’s initial length. The displacement waveform of the cantilever’s free end during beating was recorded, and the beating force profile was derived using transverse vibration analysis for cantilevers, as explained below.

The fundamental principles of transverse cantilever vibration are well documented in material mechanics textbooks [33]. The governing equation for cantilever vibration is expressed as:

(1) $$\rho A\frac{\partial^{2}y(x,t)}{\partial t^{2}}=-EI\frac{\partial^{4}y(x,t)}{\partial x^{4}}$$

where *ρ* (=10.5 g/cm^3^) is density of the silver cantilever, *A* the cross-sectional area of cantilever, *E* (=83 GPa) is elastic modulus of the cantilever, *I* the moment of inertia of *A*, *x* is the coordinate origin at the clamped end and along the length of the cantilever, *y* the coordinate orthogonal to *x*, and *t* is time.

The boundary conditions at the clamped end are:

(2a) $${\left.y(x,t)\right|}_{x=0}=0$$

(2b) $${\left.\frac{\partial y(x,t)}{\partial x}\right|}_{x=0}=0$$

At the free end, where the hooked gel exerts force, the boundary conditions are:

(3a) $${\left.y(x,t)\right|}_{x=L}=s(t)=\frac{S_{0}}{2}+\sum_{j}S_{j}\cos(\omega_{j}t+\varphi_{j})$$

(3b) $${\left.\frac{\partial^{2}y(x,t)}{\partial x^{2}}\right|}_{x=L}=0$$

Here, *L* is the cantilever length; *s*(*t*) indicates the measured beating displacement at the gel hook, and the right side in Equation (3a) is the real part of the Fourier transform of *s*(*t*).

By using variable separation and substituting the boundary conditions, we can finally obtain

(4) $$\begin{array}{l} y(x,\;t)=\sum_{j}\left(C_{1}^{j}(\cosh k_{j}x-\cos k_{j}x)+C_{2}^{j}(\sinh k_{j}x-\sin k_{j}x))\cos(\omega_{j}t+\varphi_{j}\right)\\ \mathrm{where}\;k_{j}={\left(\frac{\rho A\omega_{j}^{2}}{EI}\right)}^{\frac{1}{4}},\\ C_{1}^{j}=S_{j}\frac{\sinh k_{j}L+\sin k_{j}L}{\left(\cosh k_{j}L-\cos k_{j}L)(\sinh k_{j}L+\sin k_{j}L)-(\cosh k_{j}L+\cos k_{j}L)(\sinh k_{j}L-\sin k_{j}L\right)}\\ C_{2}^{j}=-S_{j}\frac{\cosh k_{j}x+\cos k_{j}x}{\left(\cosh k_{j}L-\cos k_{j}L)(\sinh k_{j}L+\sin k_{j}L)-(\cosh k_{j}L+\cos k_{j}L)(\sinh k_{j}L-\sin k_{j}L\right)} \end{array}$$

The shear force in the cross-section at the hook end of the cantilever *B*(*t*) is regarded as the beating force of the gel exerted to the cantilever.

(5) $$B(t)=\frac{3S_{0}EI}{2L^{3}}+{\left.\frac{1}{EI}\frac{\partial^{3}y}{\partial x^{3}}\right|}_{x=L}$$

**Figure 4:**
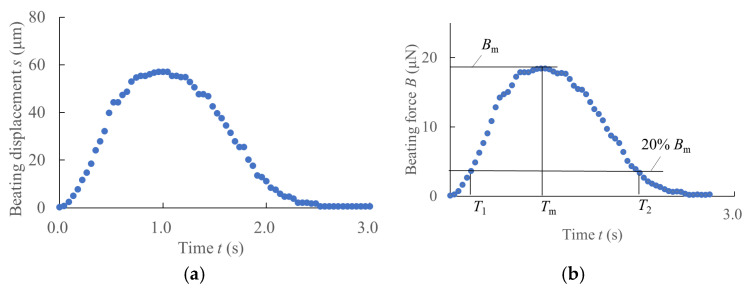
Dynamic evaluation of the beating force. (**a**) Recorded displacement at the free end of the cantilever probe during hiPS-CM beating. (**b**) Dynamic waveform of the beating force derived using the transverse forced oscillation theory for a cantilever. The evaluation parameters are annotated on the force waveform in (**b**).

Figure 4 illustrates a sample analysis. Figure 4a shows the original displacement waveform recorded at the cantilever hook, while Figure 4b presents the corresponding force profile derived using transverse vibration equations. Static loading tests were performed to calibrate the cantilever, confirming that the deflection–load relationship aligned with theoretical predictions.

Moreover, to take the advantages of the beat force dynamic profile obtained here, we measured the following time points from the force waveform (*T_m_*, *T*_1_, and *T*_2_ on Figure 4b) and defined three parameters to aid the evaluation of subtype differentiation: *T_m_* is the time when beating force reaches its maximum *B_m_*, *T*_1_ is the time as beating force increasing to 20% of *B_m_* from beating start, *T*_2_ is the time as beating force decreases to 20% of *B_m_*. Three parameters, contraction velocity *F_c_* (μN/s), relaxation velocity *F_r_* (μN/s), and force–time integral *FI* (μN·s), are defined as follows:

(6) $$F_{c}=\frac{B_{m}-0.2B_{m}}{T_{m}-T_{1}};\;\mathrm{and}\;\mathrm{define}\;\mathrm{normalized}\;F_{c}\;\mathrm{as}\;{F^{\prime }}_{c}=\frac{F_{c}}{B_{m}}=\frac{0.8}{T_{m}-T_{1}}$$

(7) $$F_{r}=\frac{B_{m}-0.2B_{m}}{T_{2}-T_{m}};\;\mathrm{and}\;\mathrm{normalized}\;F_{r}\;\mathrm{as}\;{F^{\prime }}_{r}=\frac{F_{r}}{B_{m}}=\frac{0.8}{T_{2}-T_{m}}$$

(8) $$FI=\int_{T_{1}}^{T_{2}}B(t)\;dt;\;\mathrm{and}\;\mathrm{normalized}\;FI\;\mathrm{as}\;FI^{\prime }=\frac{1}{B_{m}}\int_{T_{1}}^{T_{2}}B(t)\;dt$$

The normalized parameters *F’_c_* and *F’_r_* for atrial tissue are greater than those for ventricular tissue, whereas *FI’* of atrial tissue is smaller than that of ventricular tissue [34]. Therefore, these three parameters can be used to discriminate the extent of cardiac subtype differentiation.

### 2.5. Tensile Test of Electrode and PDMS Chamber Materials

To evaluate the mechanical compatibility between the electrode material and the PDMS used for the culture chambers, tensile tests were performed at room temperature using a uniaxial tensile test apparatus developed in our lab [35]. Membranes with a thickness of 1.0 mm were fabricated from the PEDOT/PSS-latex-rGO electrode material and PDMS, respectively. Test specimens were prepared by cutting the membranes into dumbbell-shaped pieces using a punching blade (Japanese Industrial Standard No. 7, with an overall length of 35 mm, width of 6 mm, and test section dimensions of 12 × 2 mm). Each specimen was clamped in a PBS-filled bath within the tensile test apparatus and stretched to a strain of 0.5 at a strain rate of 0.006 s^−1^.

### 2.6. Immunofluorescence Observation

The cultured gel was carefully separated from the embedded electrodes and removed from the chamber. It was fixed with 4% paraformaldehyde and permeabilized with 2% Triton X-100 for 15 min at room temperature. Nonspecific binding was blocked by incubating the gel in 3% (*v*/*v*) goat albumin in phosphate-buffered saline (PBS) for 1 h at 37 °C. Primary antibodies, including rabbit IgG anti-myosin light chain 2 (MLC2v) polyclonal (1:200, Proteintech, Rosemont, IL, USA) and mouse IgG1 anti-cardiac troponin T (cTnT) (1:200, Thermo Fisher Scientific, MA, USA), were used. Corresponding secondary antibodies, Alexa488 goat anti-rabbit IgG (1:1000, Thermo Fisher Scientific, MA, USA) and Alexa546 goat anti-mouse IgG1 (1:1000, Thermo Fisher Scientific, MA, USA) were applied. Following immunostaining, the gel was incubated with 300 nM DAPI solution (Thermo Fisher Scientific, MA, USA) for 5 min to stain the nuclei.

### 2.7. Measurement of Gene Expression

The genes investigated in this study are listed in Table 1. Gene expression was analyzed using real-time polymerase chain reaction (rtPCR), with *GAPDH* serving as the internal reference. mRNA was extracted using a conventional phenol-based method, and cDNA synthesis was performed with the PrimeScript RT reagent kit (Takara Bio, Shiga, Japan). RT-PCR was carried out on a Thermal Cycler (Takara Bio, Shiga, Japan) under the following conditions: an initial denaturation at 95 °C for 30 s, followed by 45 cycles of denaturation at 95 °C for 5 s and annealing at 60 °C for 30 s. For the expression at the end of first stage (day 15 after monolayer differentiation), relative expressions to the internal reference *GAPDH* were calculated (ΔCt method). For the expression after the second stage (3D culture with physical stimulation for 15 days), the relative expression levels of the genes were calculated using the ΔΔCt method with respect to the control (3D culture without any physical stimulation).

### 2.8. Statistical Analysis

*T*-test was applied to two-population comparisons (results on the beating force characteristics in Section 3.4). For multiple comparisons, one-way ANOVA non-parameter analysis was performed followed by the Dunnet test (BellCurve for Excel 3.20). *p* < 0.05 was regarded as significant.

## 3. Results

### 3.1. Mechanical Compatibility Between Culture Chamber and the Embedded Electrodes

**Figure 5:**
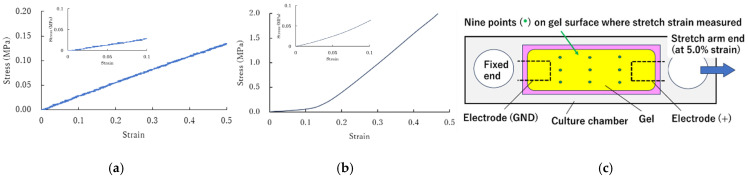
Mechanical compatibility between the culture chamber and its embedded electrodes. (**a**) Tensile stress–strain relationship of the PDMS material used for the culture chambers. (**b**) Stress–strain relationship of the elastic electrode material. (**c**) Measurement of surface stretch strain at three regions (GND, middle, and (+) side) on the cultured gel. Insets in (**a**,**b**) are the stress–strain relationships within 0–0.1 strain range.

Figure 5a,b illustrate the tensile properties of the culture chamber material (PDMS) and the electrode material, respectively. The tensile characteristics of the PDMS were linear, while the electrode material exhibited a nonlinear, strain-stiffening behavior. The elasticity of the PDMS was measured as 0.32 ± 0.05 MPa (n = 5), and the elasticity of the electrode material was 0.66 ± 0.16 MPa (n = 4) at 5% strain, corresponding to the maximum mechanical stretch applied to the cultured gels in this study. These results indicate that the culture chamber and the embedded electrodes were mechanically compatible.

To assess the strain imposed on the cultured gels, nine locations were selected on the gel surface (Figure 5c), and the maximum surface stretch strains were measured using video images captured by a microscope. The maximum strains were 5.6 ± 1.6%, 4.2 ± 2.1%, and 4.2 ± 1.5% for the regions at the GND, middle, and (+) side of the gel relative to the electrodes, respectively. These findings confirmed that the mechanical stimuli were effectively transmitted to the cultured gels through the chamber–electrode–gel configuration.

### 3.2. Coordination of Mechanical and Electrical Stimuli

**Figure 6:**
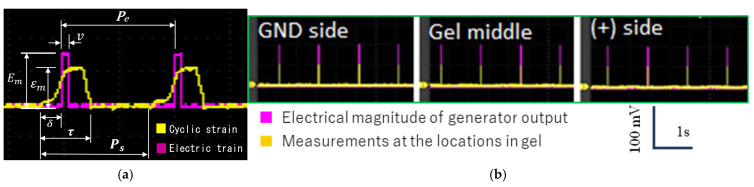
(**a**) Waveforms of the mechanical and electrical stimuli, with the parameters defining the stimulations. (**b**) Measured electrical pulse at three regions relative to the electrodes in the cultured gel.

Figure 6a shows the simultaneous application of mechanical and electrical stimuli to a gel in culture. The electrical stimulus is defined by its pulse voltage (*E_m_*), the period of the electrical train (*P_e_*), and the pulse width (*υ*). The mechanical stretch is characterized by its magnitude (*ε_m_*) and the stretch interval (τ). The mechanical stimulus period (*P_s_*) is controlled by the motor’s rotational speed (γ), and *P_e_* is identical to *P_s_* since each motor rotation triggers one electrical stimulus. The values of *ε_m_* and *τ* are determined by the design of the cam mechanism, while *E_m_* and υ are adjusted through the stimulus generator. The time phase (δ) between the mechanical and electrical stimuli is modulated by programming a delay in the electrical pulse after it is triggered. Table 2 lists the operational parameters of the bioreactor system and their respective variable ranges, as confirmed in this study.

To verify the stimulus characteristics, we measured the practical voltage at the GND, middle, and (+) sides (Figure 5c) of the cultured gel by inserting a multimeter probe. As shown in Figure 6b, the voltage magnitude at each location was approximately half of the output pulse from the generator, which is consistent with the quasi-uniform bulk potential characteristics observed in high-conductivity electrolytes [36].

### 3.3. Differentiation Status of hiPS Cells at Day 15 After the First Monolayer Differentiation Stage

**Figure 7:**
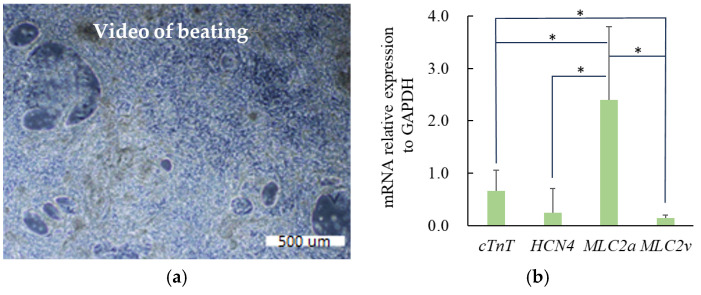
Differentiation status after first monolayer differentiation stage of 15 days. (**a**) Video clip of hiPS-CMs beating and (**b**) mRNA relative expressions with respect to the internal reference GAPDH at the end of this stage (data plotted by means and SD, n = 5; asterisk indicates *p* < 0.05).

Figure 7 illustrates the status of the hiPS-CMs after the first monolayer differentiation stage. Obvious beating of the hiPS-CMs was observed under the microscope. The expression levels of mRNAs related to cardiomyocyte subtypes (Figure 7b) indicated that the initial differentiation of cardiomyocytes at this stage was characterized by high expressions of *MLC2a* and *cTnT*, alongside low expression of *MLC2v*, consistent with the results reported in [28]. hiPS-CMs in this state were then harvested for 3D culture under integrated stimuli using the bioreactor. Video of hiPS-CMs beating in Supplementary Materials.

### 3.4. Results on the 3D Culture of hiPS-CMs Populated in vECM-Collagen Gels and Stimulated at Low (1.0 Hz) and High (5.0 Hz) Frequencies

**Figure 8:**
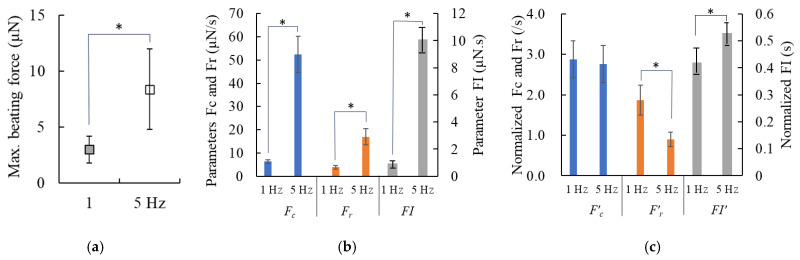
Dynamic evaluation of beating force under mechanical and electrical stimulation at low (1.0 Hz) and high (5.0 Hz) frequencies. (**a**) Maximum beating force *B_m_*, (**b**) contraction velocity *F_C_*, relaxation velocity *F_r_*, and force-time integral *FI*, and (**c**) corresponding normalized parameters *F’_C_*, *F’_r_*, and *FI’*. (data plotted as mean ± SD, two experiments were conducted and n = 3~5 beating force waveforms were analyzed for each experiment; asterisk indicates *p* < 0.05).

**Figure 9:**
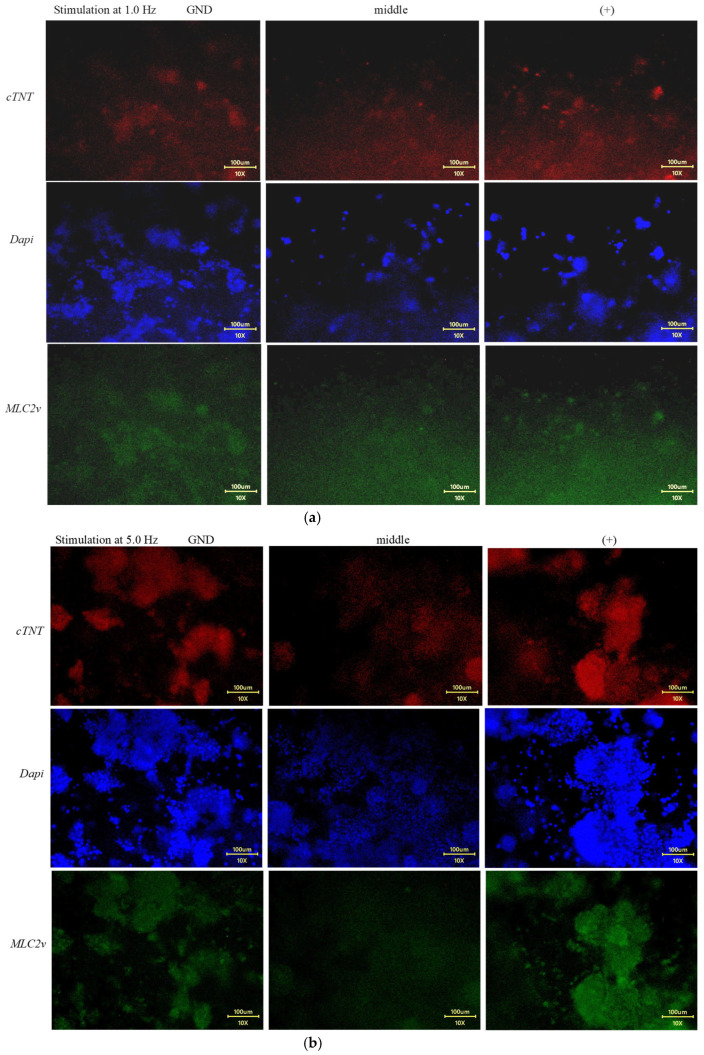
Observation of *cTnT*, DAPI, and *MLC2v* expression in cells cultured at different portions of gels relative to the electrodes under stimulation at frequencies of 1.0 Hz, nine panels in (**a**) and of 5.0 Hz, nine panels in (**b**). It should be kept in mind that, since these images were taken within the 3D hydrogels, the resolution was quite compromised—particularly when the target markers were less expressed, as seen in (**a**).

**Figure 10:**
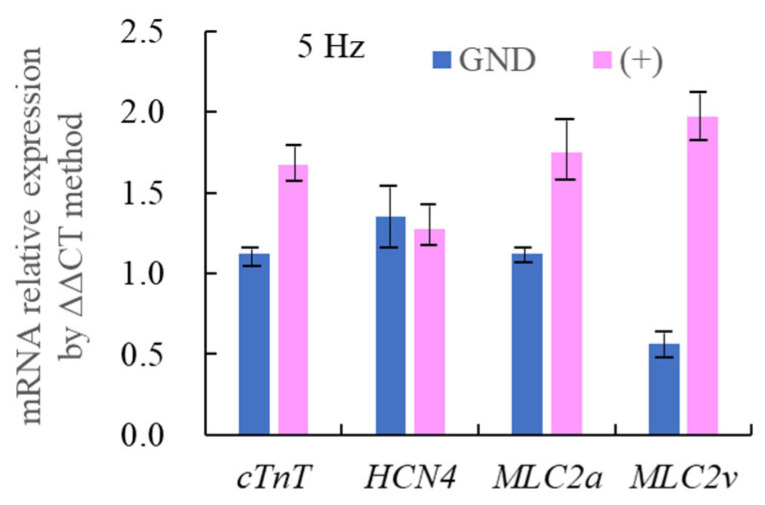
Relative mRNA expression of at GND and (+) sites under 5.0 Hz stimulation. Expressions were analyzed using the ΔΔCT method. The control group consisted of 3D cultures in vECM-collagen hydrogels without any physical stimulation. (n = 2, with two measurements and their average shown for each result).

The values of the operational parameters for this experiment are listed on the far-right side of Table 2. Figure 8, Figure 9 and Figure 10 present the results of hiPS-CMs cultured in vECM-collagen gels under mechanical and electrical stimulation at 1.0 Hz and 5.0 Hz, respectively. Figure 8a–c show the beating force characteristics. It can be observed that stimulation at 5.0 Hz significantly enhanced the beating force compared to 1.0 Hz stimulation (Figure 8a). The beating force increase resulted in significant elevations in parameters Fc, Fr, and FI (Figure 8b). However, a comparison of the corresponding normalized quantities of these parameters under 1.0 Hz and 5.0 Hz stimulations showed that the normalized contraction velocity *F’_c_* and relaxation velocity *F’_r_* actually decreased while normalized force–time integral *FI’* remained increased (Figure 8c), which indicated that the beating behavior under 5.0 Hz stimulation shifted toward a more ventricular-like pattern. The maximum beating force *B_m_* and parameters *F_c_*, *F_r_*, and *FI* of the gels under 1.0 Hz stimulation did not show significant changes compared to those of gels cultured without any physical stimulation.

Immunofluorescence observation (Figure 9a) of the gels under 1.0 Hz stimulation did not reveal any significant features. Cells were roughly uniformly distributed across the GND, middle, and (+) sides of the gels, and the expressions of *cTnT* and *MLC2v* were maintained at low levels at each location. In contrast, gels under 5.0 Hz stimulation (Figure 9b) showed an interesting impact from the stimulation. Cardiac markers (*cTnT* and *MLC2v*) were upregulated at the electrode sides, GND and (+), compared to the 1.0 Hz stimulation. In particular, cells on the (+) electrode side exhibited the most intense *MLC2v* expression. Meanwhile, cells in the middle part of the gels showed lower levels of DAPI and cardiac expression.

To further investigate this location-dependent phenomenon, we examined cardiac mRNA expression at the GND and (+) portions of the gels under 5.0 Hz stimulation, respectively. Compared to Figure 7b, which represents the differentiation status of cells at the beginning of 3D culture with physical stimulation, Figure 10 shows relative elevations in *HCN4* and *MLC2v* expressions and relative reductions in *MLC2a* expression compared to *cTnT* at both electrode sites. This indicates the progression of cardiac differentiation, as previously reported [6,14,27].

A key finding of this study was the electrode-site-dependent cardiac differentiation. With reference to non-stimulated control samples, Figure 10 shows that cardiac differentiation markers (*cTnT*, *MLC2a*, and *MLC2v*) were substantially upregulated on the (+) electrode side, whereas *MLC2v* was notably repressed on the GND side. The mRNA expression of these markers under 1.0 Hz stimulation showed no evident differences compared to control samples at both electrode sites. Thus, Figure 10 also implies the differences in mRNA expression between samples stimulated at 5 Hz and those at 1 Hz.

## 4. Discussion

Cardiac differentiation is one of the earliest processes in human organogenesis. However, cardiac maturation extends over a much longer period, spanning the neonatal phase and undergoing dynamic changes, including variations in oxygen levels and embolic substrates. These changes are accompanied by feedback from the tissue’s contractile development itself [5,37]. These characteristics of cardiac development have led to the current challenges in applications of cardiomyocytes differentiated from hiPS cells. While cardiac commitment is relatively straightforward, achieving adult-like maturation remains highly challenging. This limitation is reflected in the clinical application of hiPS-CMs, where no functional cardiac pump activity can currently be achieved. Closely linked to the maturation challenge is the differentiation of cardiac subtypes, a process that is heavily influenced by cardiac maturation (via enhancement or selection at the progenitor stage) rather than commitment [38].

To date, it has been reported that physical stimulations, biochemical factors, functional culture substrates, and three-dimensional culture approaches are particularly effective in promoting the maturation of differentiated CMs. However, most of those culture systems could only impose single types of stimulations to cultured cells. The most impressive and manifest effect of the stimulations is the enhancement of cardiac maturation in terms of the increased beating force and cardiac tissue-like structure [6,14,39]. However, when considering the phenotypic spectrum of cardiac gene expression and cardiac subtype differentiation [38,40,41], researchers still face many challenges to overcome. Given the complexity and sophistication of cardiac subtype differentiation and maturation processes, the optimal strategy likely involves integrating multiple effective factors rather than relying on any single approach. Studies have shown that as individual stimulatory factors are introduced into the hiPS-CMs culture system, the level of CM maturity improves with increasing complexity of the engineered tissues [41,42]. Supporting this integrative approach, evidence suggests that each stimulation factor exerts its enhancing effects relatively independently [21]. This means that combining multiple stimuli produces cumulative positive effects, surpassing or without the risk of adverse interactions between them.

Building on these findings, we developed this integrated bioreactor system aimed at exploring the differentiation and maturation of hiPS-CMs across much broader aspects, particularly in cardiac subtype differentiation, by introducing multiple stimuli and cues. The preliminary results presented here are encouraging. The system enables the coordinated application of mechanical, electrical, and substrate biochemical stimuli in a 3D culture environment. A notable feature of this system is its ability to adjust the magnitudes of mechanical and electrical stimuli, as well as their temporal synchronization. Additionally, we conducted a dynamic analysis of the beating force generated by 3D hiPS-CMs constructs and introduced three time parameters related to beating dynamics to evaluate subtype differentiation. This dynamic evaluation represents an advancement over current methods, which rely on static extrapolations of contractile force [43].

A compelling outcome of our preliminary experiments using this system is the location-dependent appearance of cardiac subtypes under high-frequency stimulation. Two potential explanations for this phenomenon are as follows: (1) the direct impact of stimulation on cells in situ, and (2) the migration of different subtypes induced by the stimulation. At the macroscale level, no significant differences were observed among the GND, middle, and (+) electrode sides, as the mechanical stretch strains were measured at 5.6 ± 1.6%, 4.2 ± 2.1%, and 4.2 ± 1.5%, respectively; the electrical pulse intensities at these locations were approximately half the generator’s output. However, at the microscale level, the cellular environment near the electrodes likely differs due to opposing polarity. Notably, the lower cellular density observed at the middle site, indicated by reduced DAPI and *cTnT* expressions, suggests that cellular migration may play a key role in this location-dependent phenomenon. This is because no evident micro- or macrolevel factors may suggest significant cellular death specifically at the middle site. If cellular death had occurred, it would be more likely at the electrode sites.

Both electrical field and mechanical stimulations are well-recognized cues for cellular migration [44,45]. Electrotaxis and chemotaxis, for instance, share overlapping signaling pathways involving TORC2 and PI3K/PTEN, which regulate front–rear polarity [45]. As for the physical driving forces behind cellular migration, recent studies on epithelial MDCKII cells have identified mechanisms involving the interplay between cellular adhesion and contractility [46]. This may suggest that ventricular cells, with their strong contractile and adhesion properties, can undergo self-sorting under electrotactic stimulation. Another reason regarding the plausible cellular migration in our experiments should also be pointed out. The 15-day differentiated hiPS-CMs possessed weak collagen compaction ability as cultured in the 3D collagen lattice, and the addition of vECM to the 3D gels, interestingly, further diminished gel compaction. As a result, no significant gel compaction occurred during the 3D culture. Consequently, the vECM-collagen hydrogels were maintained at a relatively low concentration (2.1 mg/mL) throughout the culture, which facilitated the migration and aggregation of hiPS-CMs towards electrode sides.

The limitations of this study are clear. The full capacity of the bioreactor has yet to be explored. In particular, further investigation is needed into the effects of varying magnitudes of mechanical and electrical stimuli, as well as the timing phase (δ in Figure 6a) between these stimuli. The observed location-dependent subtype appearance may offer a valuable approach for advancing subtype-specific cardiac maturation and separation. However, the available data in this study remain limited; for instance, only two experiments were conducted as we obtained the data in Figure 8, although the significance test was performed using multiple waveforms in each measurement. For our ongoing research, we aim to conduct additional experiments to strengthen the supporting data and to utilize live-cell fluorescence imaging (e.g., Fluo-4 AM and TMRM dyes) to track the potential migration of cardiomyocytes within the gels. This will help us further elucidate the underlying mechanisms of this phenomenon and enhance its effects. 

## Figures and Tables

**Table 1:** Information for the test of mRNAs by means of real-time polymerase chain reaction (rtPCR).

mRNA	Primers	Product Length (bp)	Indication of Expression
*GAPDH*	Sense: 5′-GTGGACCTGACCTGCCGTCT-3′ Antisense: 5′-GGAGGAGTGGGTGTCGCTGT-3′	153	Housekeeping
*cTnT*	Sense: 5′-AGCGGAAAAGTGGGAAGAGG-3′ Antisense: 5′-GGTCGAACTTCTCTGCCTCC-3′	165	Overall cardiac cells
*HCN4*	Sense: 5′-AGCATGGTCAGGTCCCAGTA-3′ Antisense: 5′-CCCTGTGCCAATCTCCACAT-3′	192	pacemaker
*Mlc2a*	Sense: 5′-CCGTCTTCCTCACGCTCTT-3′ Antisense: 5′-TGAACTCATCCTTGTTCACCAC-3′	120	atrial cell
*Mlc2v*	Sense: 5′-TACGTTCGGGAAATGCTGAC-3′ Antisense: 5′-TTCTCCGTGGGTGATGATG-3′	138	Ventricular cell

**Table 2:** Parameters for the bioreactor system operation.

Parameters (Unit)	Symbols	Variable Range	Values in the Experiment
Rotation speed of the motor (rotation per second)	*γ*	0.5~10	1.0 and 5.0
Frequency of mechanical stretch (Hz)	*fm*	=*γ*	1.0 and 5.0
Period of stimulus interval (s)	*Ps*	=1/*γ*	1.0 and 0.2
Width of the mechanical stretch (s)	*τ*	≈0.5 *Ps*	0.5 and 0.1
Maximum stretch strain (-)	*ε* *_m_*	0.01~0.10	0.05
Electrical pulse amplitude (mV)	*Em*	10~10,000	100
Period of electrical pulse (s)	*Pe*	=*Ps*	1.0 and 0.2
Frequency of the electrical pulse (Hz)	*fe*	=*fm*	1.0 and 5.0
Width of the electrical pulse (ms)	*υ*	0~*Pe*	1.0
Phase between the two stimuli (ms)	*δ*	0~*Ps*	0

## Data Availability

The datasets generated during and/or analyzed during the current study are available from the corresponding author on reasonable request.

## Supplementary Materials

The following supporting information can be downloaded at: https://www.mdpi.com/article/10.3390/jcdd12020056/s1, Video S1: The beating of hiPS-CMs on day 15 after the initiation of cardiac differentiation.
